# The M. Powell Lawton Award Lecture and Presentation

**DOI:** 10.1093/geroni/igaf122.1919

**Published:** 2025-12-31

**Authors:** Christine Mueller

## Abstract

The M. Powell Lawton Award Lecture will feature an address by the 2024 recipient Stephen M Golant, PhD, FGSA, of the University of Florida. This session will also include the presentation of the 2025 M. Powell Lawton Award to recipient Joseph E. Gaugler, PhD, FGSA. The M. Powell Lawton Award is presented annually to an individual who has made outstanding contributions from applied research that has benefited older people and their care. The Lawton Award is generously funded by the Polisher Research Institute of Abramson Senior Care.

